# Healthy Body Image Intervention Delivered to Young Women via Facebook Groups: Formative Study of Engagement and Acceptability

**DOI:** 10.2196/resprot.9429

**Published:** 2018-02-20

**Authors:** Jerod L Stapleton, Sharon L Manne, Ashley K Day, Kristine Levonyan-Radloff, Sherry L Pagoto

**Affiliations:** ^1^ Rutgers Cancer Institute of New Jersey Department of Medicine Rutgers University New Brunswick, NJ United States; ^2^ Rutgers Cancer Institute of New Jersey New Brunswick, NJ United States; ^3^ Institute for Collaboration on Health, Intervention and Policy University of Connecticut Storrs, CT United States

**Keywords:** body image, dissonance-based intervention, indoor tanning bed, social media, Facebook, behavioral intervention, prevention

## Abstract

**Background:**

There is increasing interest in using social media sites such as Facebook to deliver health interventions so as to expose people to content while they are engaging in their usual social media habit. This formative intervention development study is novel in describing a preliminary test of using the secret group feature of Facebook to deliver a behavioral intervention targeting users of indoor tanning beds to reduce their risk of skin cancer. Intervention content was designed to challenge body image-related constructs associated with indoor tanning through the use of dissonance-inducing content.

**Objective:**

To evaluate engagement with and acceptability of using a secret Facebook group to deliver a healthy body image intervention to young women engaged in indoor tanning.

**Methods:**

Seventeen young women completed a baseline survey and joined a secret Facebook group with intervention content delivered via daily posts for 4 weeks. Engagement data was extracted and acceptability was measured via a follow-up survey.

**Results:**

The study had a high retention rate (94%, [16/17]). On average, posts were viewed by 91% of participants, liked by 35%, and commented on by 26%. The average comment rate was highest (65%) for posts that elicited comments by directly posing questions or discussion topics to the group. Average intervention acceptability ratings were highly positive and participants reported feeling connected to the group and its topic. Average rates of past 1-month indoor tanning reported following the intervention were lower than the baseline rate (*P*=.08, Cohen d=0.47).

**Conclusions:**

This study is novel in demonstrating participant engagement with and acceptability of using Facebook secret groups to deliver a dissonance-inducing intervention approach that utilizes group-based discussions related to body image. The study is also unique within the field of skin cancer prevention by demonstrating the potential value of delivering an indoor tanning intervention within an interactive social media format. The findings suggest that Facebook metrics of intervention post engagement (ie, likes and comments) may vary based on post types and that designing specifically labeled discussion posts may be helpful for soliciting engagement as well as challenging beliefs.

## Introduction

Social media allows for the unlimited exchange of information, images, and interactive peer communication. Researchers have begun to utilize Facebook, the most popular social media site [1-3], to deliver health interventions so as to expose people to content while they are engaging in their usual social media habit [4]. This proof-of-concept study examined the use of Facebook to deliver intervention content designed to reduce indoor tanning bed use among young women. The intervention utilized persuasive techniques to shift unhealthy attitudes and perceptions linked to indoor tanning toward healthier perspectives. Indoor tanning is linked to an increased risk of melanoma [5] and is implicated as a central contributor to rising melanoma rates among young US women [6].

The intervention approach is guided by body image theories that demonstrate cultural experiences can negatively impact how young women view their bodies and may lead to a preoccupation with obtaining an unrealistic, culturally-derived ideal appearance [7,8]. The negative body image and body dissatisfaction associated with endorsing these appearance ideals represent core motives for a variety of unhealthy appearance-oriented behaviors, including use of tanning beds [9]. Efficacious behavioral health interventions (eg, [10,11]) have targeted risky body image beliefs through the use of persuasive techniques based in cognitive dissonance theory. These techniques encourage participants to engage in cognitive exercises and discussions during which they endorse, and ultimately adopt as their own, attitudinal perspectives that are counter to and conflict with their unhealthy ideals and negative body image beliefs [12]. The conflict between these counter perspectives and held beliefs leads to psychological discomfort (ie, cognitive dissonance) [12]. The person is then motivated to seek psychological relief by altering their original unhealthy beliefs to be more consistent with the healthier perspective being advocated. Adapting such healthier perspectives can lead to reductions in body dissatisfaction and resulting risk behavior such as tanning. Dissonance-based interventions are often delivered within small in-person groups since engaging with counter-attitudinal information in a group format increases the amount of dissonance experienced and leads to a greater likelihood of attitude and behavior change [12,13]. Social media features such as “secret” (ie, private) Facebook groups are designed to share information and generate conversations among groups of users with common interests [14]. Social media represents an intriguing but unexplored approach to delivering these interventions given features that easily connect groups of people.

The purpose of this proof-of-concept study was to test engagement with and acceptability of delivering a dissonance-based, body image focused intervention to reduce indoor tanning using Facebook secret groups. For this intervention, content was posted daily on the group page for four weeks and group members were encouraged to share and discuss their opinions about the posts. We also examined the preliminary efficacy of the intervention by comparing rates of participants’ tanning bed use before and after the intervention.

## Methods

### Participants

Participants were 18 to 25-year-old women who used Facebook at least once a day and a tanning bed at least once in the past year. Recruitment utilized multiple methods including: emailing study invitations to participants from our prior focus group study on tanners’ intervention preferences; distributing recruitment flyers to students on a large northeastern US college campus; posting study flyers on social media accounts (including investigators’ personal Facebook and Twitter pages); and ads on local Craigslist pages. Research staff conducted study screening phone calls with interested participants to evaluate study eligibility. A total of 17 participants were enrolled (6 recruited from prior focus groups, 3 campus flyers, 6 social media and Craigslist, and 2 referrals from enrolled participants).

### Procedures

Study procedures included a baseline survey, participation in a four-week Facebook group, and a post-intervention survey 5 weeks after baseline. The study was conducted from July to August 2016. Enrolled participants were emailed a link and a unique PIN to complete the online baseline survey. Participants received a US $30 gift card for each survey and those who completed both were entered in a US $100 gift card raffle. The university’s Institutional Review Board approved all study procedures and participants provided online consent.

### Intervention

The intervention consisted of a secret invitation-only Facebook group named “RU Facebook Project”. The group feature of Facebook is designed to connect and share information with a subset of Facebook users based on shared interests. With Facebook secret groups, Facebook users receive a private invitation to join a group, which in this study was used to post the intervention messages. Further, secret group membership and content is limited to invited group members and their group membership and in-group activities are not publicly viewable to outside Facebook users. A study Facebook account was created and used to deliver all intervention content via daily group posts. Two authors, JS and AD, joined the group with study-specific Facebook user accounts to provide a “face” for the researchers and increase study credibility. Our involvement was purposefully limited to include: encouraging initial comments and responses to discussion questions by JS and AD—commenting once each on the first “icebreaker” post and AD commenting once on each of the next two discussion posts—and liking comments from participants throughout the study to reinforce participation.

The goals of the dissonance-based Facebook intervention approach mirrored those of existing interventions for disordered eating and indoor tanning [11,15-17] including: raising awareness and promoting reflection of sociocultural and media influences on body image and risk behaviors; 2) promoting dissonance by encouraging participants to speak out against idealistic thinking and endorse counter perspectives by commenting on the group Facebook page; and 3) encouraging body acceptance. The process of creating Facebook messages involved developing a posting strategy to cover the type and range of content typically provided within group-based disordered eating interventions [15-17] and our website tanning intervention [11]. This process resulted in four posting categories, or posting types, designed to specifically address each goal (Table 1). JS wrote or adapted posts designed to address each goal and refined them based on feedback from other study authors. Several of the *Your Thoughts* posts, the primary approach for encouraging dissonance processing, were adapted from group-based discussion questions from prior disordered eating interventions. Content for inspirational posts were primarily procured from popular media sources including Facebook. Most posts (93%) were written to generally focus on body image and women’s experiences rather than specifically addressing indoor tanning in order to reduce possible reactance to content from tanners while still addressing an important predictor of tanning behavior. There was no predetermined order of presentation of post types and we varied their order across the 4-week intervention period to avoid making repetitive post types across multiple days. Your Thoughts discussion posts were made every 2-3 days and the homework posts were made in weeks 3 and 4 as they were designed to have group members apply knowledge learned earlier in the intervention period.

### Intervention Receipt and Engagement Metrics

Intervention receipt was defined as the percent of posts viewed by participants. Engagement was assessed as interactions with group posts (ie, likes and comments). Engagement results are presented both as “post engagement”, defined as the mean number of likes and comments received by post types, and “participant engagement”, defined as the number of likes and comments averaged across participants. Facebook data was manually extracted from the group newsfeed after the posting period ended.

### Intervention Acceptability Measures

Intervention acceptability was measured on the follow-up survey in two ways. First, four general intervention evaluation items assessed the extent participation in the Facebook intervention was interesting, understandable, useful, or positive (measured on an 11-point scale anchored with 0= *not at all* and 10= *extremely*) [11,20].

**Table 1:** Description of intervention content delivered in Facebook group posts.

Description of content within each post type	Number (total %)	Source(s)	Example post
Information-based content intended to provide context for the intervention content and counter-perspectives to idealistic thinking. Designed to raise participants’ awareness of their thoughts, feelings, and actions with regard to their appearance, including the sociocultural experiences that lead to an overemphasis on appearance and cause body dissatisfaction.	9 (32%)	Adapted from disordered eating [10,15-17] and indoor tanning intervention research [11] as well as popular sources.	“We’ve talked about the excessive use of Photoshop to create images of ‘ideal’ women by making body parts thinner or changing the appearance of skin by smoothing wrinkles, removing blemishes, and often altering skin tone to appear tanner. One reason that the use of Photoshop is so common is that images of ‘ideal’ women are used to sell products. For example, fashion magazine covers, articles, and images are designed to make a woman feel bad about her looks. These magazines try to convince women that something is wrong with how they look and that they can fix the problem by buying the products in the magazine ads. The worse the images make women feel, the more money the magazine makes in advertising sales.” This post included a link to a web article showing celebrity photographs before and after they were altered with Photoshop.
Inspirational or humorous quotes related to resisting idealistic thinking, empowerment, or body activism.	9 (32%)	Images curated from internet searches or sharing postings from other Facebook pages, for example [18,19].	A meme with a quote from Tina Fey describing her view on the female body image.
Questions or discussion topics that elicited comments and responses from group members. Posts were clearly identified with the label: “Your Thoughts **Please read and comment**”. Designed to encourage comments that were critiques of or counterarguments against unrealistic beauty ideals.	8 (29%)	Adapted from disordered eating [10,15-17] and indoor tanning intervention research [11]	“Several members have mentioned the “ideal” for women in their comments and now we would like to define the ideal to understand exactly what we are talking about. What are we told that the ideal or perfect woman looks like?”
Skill-building, homework-type activities were programmed with online survey software. Appeared as a webpage accessed with an outside link from Facebook posts. Given the personal nature of some questions, participants’ responses to homework were not directly viewed by the group. Designed to promote media literacy and self-acceptance.	2 (7%)	Adapted from disordered eating [10,15-17] and indoor tanning intervention research [11]	A positive body image task that asks participants to create a top ten list of their own best attributes.

Second, participants answered several Facebook-specific evaluation items related to their perceptions of their experience as members of the Facebook group [21-23] that included perceived connectedness with group, identification with posts and other group members, enjoyment, ease of participation, and willingness to continue to engage with the group. (measured on 5-point Likert-type scales anchored with 1= *strongly disagree* and 5= *strongly agree*)

### Preliminary Outcome Measure

Number of past 1-month tanning sessions was measured on the baseline and follow-up assessment using an expert-recommended survey item (“How many times in the past month have you used a tanning bed or booth?”) [24].

### Data Analysis

Descriptive statistics are presented for the Facebook engagement metrics and the intervention acceptability measures. A paired-sample 2-tailed t-test was used to compare mean differences in baseline and follow-up responses to the preliminary indoor tanning outcome measure.

## Results

### Participant

Participants reported a mean age of 20.8 years (SD 1.7) and 9 out of 17 (53%) were White, 5 out of 17 (29%) were other/multiracial, 2 out of 17 (12%) were Asian, and 1 out of 17 (6%) refused to answer. Five out of seventeen participants identified as Hispanic (29%). Most participants were currently enrolled in college (82% [12/17]) with the remaining having a bachelor's degree or higher (18% [3/17]). Eleven out of seventeen (65%) participants used Facebook multiple times a day and 5 out of 17 (29%) reported daily use at baseline.

### Retention

All enrolled participants completed the baseline assessment and accepted our invitation to join the Facebook group. Sixteen out of seventeen participants completed the post-intervention survey, producing a 94% study retention rate.

### Intervention Receipt

Twelve out of seventeen participants (70.6%) viewed every post and an additional four (23.5%) viewed at least 75% of posts. An average of 91.4% participants viewed each post (See Table 2) and the mean post view rate was similar across each post type.

### Engagement

Table 2 also presents post engagement averaged across the posting period. Posts were liked on average by 34.6% (SD 21.1) of participants with the highest like rates for inspirational (50.3% [SD 12.5]) and psycho-educational (42.5% [SD 11.3]) posts. An average of 26.2% (SD 28.7) of participants commented on a typical post with the highest rates for Your Thoughts posts (64.7% [SD 14.4]). Nearly half of participants (41.2%) commented within the webpages that were linked to in the homework posts. For participant-level engagement, participants, on average, liked 9.1 posts (SD 6.1) and commented on 7.6 (SD 4.1).

**Table 2:** Descriptive statistics for post views and engagement metrics by post type.

Engagement Metric and Post Type		Mean percentage (SD)
**Views**		
	All^a^	91.4 (7.1)
	Psycho-educational	90.2 (7.2)
	Inspirational	89.5 (7.1)
	Your Thoughts	94.9 (6.6)
**Likes**		
	All	34.6 (21.1)
	Psycho-educational	42.5 (11.3)
	Inspirational	50.3 (12.5)
	Your Thoughts	8.1 (7.7)
**Comments**		
	All	26.2 (28.7)
	Psycho-educational	13.0 (14.3)
	Inspirational	5.2 (4.6)
	Your Thoughts	64.7 (14.4)

^a^Homework posts are not included in descriptive statistics given the low number of posts relative to other post types and the nature of the posts that contained links to an external webpage for participants to leave comments.

**Table 3:** Perceptions of the Facebook group intervention experience. Survey items were measured with a 5-point, Likert-type response scale: 1= *strongly disagree*, 2= *disagree*, 3= *neither,* 4= *agree*, 5= *strongly agree*.

Survey Type and Items			Mean (SD)
**Connection with intervention and group**			
	I could identify with a lot of the posts.	4.3 (0.6)	
	The posts were relevant to me.	4.4 (0.5)	
	I could identify with other people in the group.	4.3 (0.6)	
	I felt connected to the other people in the group.	3.9 (0.7)	
	I paid attention to other people’s comments in the group.	4.4 (0.6)	
	I felt like I was actively involved in the Facebook group.	4.2 (0.8)	
**Perceptions of the Facebook group experience**			
	I enjoyed expressing my opinions in the group.	4.2 (1.0)	
	I enjoyed reading other people’s comments in the group.	4.2 (0.7)	
	I enjoyed reading the posts made in the Facebook group.	4.4 (0.5)	
	I felt comfortable participating in the study.	4.6 (0.8)	
	It was easy to participate in the Facebook study.	4.8 (0.5)	
	The study was too time consuming.	2.2 (1.3)	
	I would be willing to continue as part of the Facebook group if the study were to continue.	4.8 (0.5)	

### Intervention acceptability

Participants provided favorable ratings on general intervention evaluation items including: interesting (mean 7.5 [SD 1.7]); understandable (mean 9.1 [SD 1.3]); useful (mean 8.2 [SD 1.7]); and positive (mean 9.1, [SD 1.5]). Means for Facebook-specific evaluation items indicated a general level of agreement with regard to perceived connectedness to the group (Table 3) including items that assessed: identification with posts (mean 4.3 [SD 0.6]), identification with other group members (mean 4.3 [SD 0.6]), connectedness to the group (mean 3.9 [SD 0.7]), and perception of active involvement with the group (mean 4.2 [SD 0.8]). Participants also indicated high ratings on items of enjoyment with various aspects of the group and comfort in participation (mean 4.6 [SD 0.8]). Participants indicated they would be willing to continue as a part of the Facebook group if the study were to continue (mean 4.8 [SD 0.5]).

### Preliminary Outcomes

The mean number of reported past 1 month indoor tanning sessions was lower at the post-intervention assessment (mean 0.7 [SD 2.3]) compared to baseline (mean 2.3 [SD 4.4]) although this difference was not significant at the α=.05 level (t_15_=1.90, *P*=.08: Cohen d=0.47).

## Discussion

Intervention receipt was high as a typical post was viewed by 91.4% of participants. In addition, the majority of participants (70.6% [12/17]) viewed every post, and an additional 23.5% [4/17] viewed at least 75% of posts. Post engagement, measured by likes and comments, differed according to post type. Posts specifically designed to elicit comments by posting questions and discussion topics to the group (ie, those titled, *Your Thoughts*) received higher rates of comments than other post types. The labeling of posts as discussion posts may be helpful for soliciting engagement as well as challenging beliefs. For participant-level engagement, mean number of likes was slightly higher than comments. Overall, the observed rates of intervention engagement compare favorably to Facebook interventions on other topics (eg, physical activity, weight loss, tobacco cessation) [25-28].

Ratings on general measures of intervention acceptability (eg, interesting) were favorable and comparable to other tanning interventions delivered via booklet or website [11,20]. Facebook-specific acceptability measures were highly favorable including the perception the group allowed for social connection and self-expression, both key reasons people use social media [22]. These intervention features, along with the focus on positive body image, may increase participants’ interest and lead to a more impactful intervention. Favorable scores were also reported for enjoyment, comfort, ease of participation, and willingness to continue in the group. The study also demonstrates that participants may engage in an intervention with minimal input or guidance from moderators, an important feature likely to lead to a more sustainable intervention with greater potential for dissemination. Overall, the study expands the literature of social media interventions to include tanning and body image among young women. No significant change in past month indoor tanning was observed, however this formative study was not adequately powered to detect changes. Our next step is to conduct a fully powered efficacy trial to assess the ability of Facebook group approach to positively influence body image and reduce indoor tanning.

This formative study has some notable limitations. First, the study had a single arm design, small sample size, and was not fully powered to detect behavior change or examine psychosocial intervention mediators. Second, the sample was primarily recruited from a single geographic area using specific inclusion criteria that reduce generalizability of the findings. Our discussion posts were adapted from in-person, group-based disordered eating interventions that utilize discussion questions designed specifically to generate counter-attitudinal responses to challenge image ideals [12] (eg, “Describe your biggest pet peeve/complaint with the media, the fashion industry, or social media”). Although it is promising that a large percent of participants engaged with the discussion posts by commenting, we did not code the comments for counter-attitudinal content and the design of this formative study did not allow for a formal test of cognitive dissonance as an intervention mediator. The general lack of research in this area results in a dearth of empirical support that using dissonance-based discussions in social media groups can lead to counter-attitudinal responses although research suggests such discussions on Internet-based forums can be efficacious [10]. Thus, the question of whether the intervention is working as designed by encouraging the generation of counter-attitudinal information through Facebook group discussions and promoting cognitive dissonance among group members needs to be examined in future research. Finally, some participants (35% [6/17]) were recruited from our prior focus group research study, which may have biased responses in a positive manner.

In conclusion, social media represents an unexplored platform for delivering dissonance-based interventions for disordered eating and indoor tanning. The current formative study demonstrates the feasibility and acceptability of using Facebook as a delivery mechanism for these types of interventions. The use of Facebook groups to deliver dissonance-based interventions capitalizes on features key to the success of these interventions including group-based discussions and peer support. Embedding the intervention into the target population’s social media routine facilitates both engagement and reaches with less expense than in-person groups and developing websites incurs. The utilization of Facebook groups for delivering group-based interventions is appealing given the potential for creating highly sustainable intervention approaches with strong potential for dissemination across multiple geographic locations.
